# Chinese and Belgian pediatricians’ perspectives toward pediatric palliative care: an online survey

**DOI:** 10.1186/s12904-024-01436-0

**Published:** 2024-04-23

**Authors:** Yajing Zhong, Alice Cavolo, Veerle Labarque, Bernadette Dierckx de Casterlé, Chris Gastmans

**Affiliations:** 1 Centre for Biomedical Ethics and Law, Faculty of Medicine, KU Leuven, Kapucijnenvoer 7, Leuven, 3000 Belgium; 2 Institute of Biomedical Ethics and History of Medicine, Faculty of Medicine, University of Zurich, Zurich, Switzerland; 3 Centre for Molecular and Vascular Biology, Faculty of Medicine, KU/UZ Leuven, Leuven, Belgium; 4 Academic Centre for Nursing and Midwifery, Faculty of Medicine, KU Leuven, Leuven, Belgium

**Keywords:** Pediatrician, Pediatric Palliative Care, Chinese, Flemish, Perspective

## Abstract

**Background:**

As pediatricians play a vital role in pediatric palliative care (PPC), understanding their perspectives toward PPC is important. PPC is established for a long time in Belgium, but has a shorter tradition in China, although it is growing in the last decade. Sampling and comparing the perspectives of these pediatricians could be insightful for both countries. Therefore, we sampled and compared perspectives of pediatricians in China and Belgium toward PPC, and explored factors influencing their perspectives.

**Methods:**

We conducted a cross-sectional online survey using the validated Pediatric Palliative Care Attitude Scale (PPCAS). Over a five-month period, we recruited pediatricians practicing in China (C) and Flanders (F), Belgium. Convenience sampling and snowballing were used. We analyzed data with descriptive statistics, and evaluated group differences with univariate, multivariate and correlation tests.

**Results:**

440 complete surveys were analyzed (F: 115; C: 325). Pediatricians in both regions had limited PPC experience (F: 2.92 ± 0.94; C: 2.76 ± 0.92). Compared to Flemish pediatricians, Chinese pediatricians perceived receiving less unit support (F: 3.42 ± 0.86; C: 2.80 ± 0.89); perceived PPC less important (F: 4.70 ± 0.79; C: 4.18 ± 0.94); and faced more personal obstacles while practicing PPC (F: 3.50 ± 0.76; C: 2.25 ± 0.58). Also, select socio-demographic characteristics (e.g., experiences caring for children with life-threatening condition and providing PPC) influenced pediatricians’ perspectives. Correlational analyses revealed that pediatricians’ PPC experiences significantly correlated with perceived unit support (ρ_F_ = 0.454; ρ_C=_0.661).

**Conclusions:**

Chinese pediatricians faced more barriers in practicing PPC. Expanding PPC experiences can influence pediatricians’ perspectives positively, which may be beneficial for the child and their family.

**Supplementary Information:**

The online version contains supplementary material available at 10.1186/s12904-024-01436-0.

## Background

Over 30% of the global population are children (1–18 years old) [1]. According to the Lancet Commission, 2.5 million children die every year due to life-threatening diseases such as cancer [2]. Despite advancements in modern medicine [3], many children suffer from the severe consequences of their life-threatening medical condition [4–6]. For these patients, curative treatment may not be in their best interest because of the limited benefits, and any small benefit fails to offset the pain related to their condition and/or associated therapies [7, 8]. Therefore, providing pediatric palliative care (PPC) is warranted, as it can improve the quality of life of these vulnerable children and their families [9–12].

PPC is comprehensive care that eases physical suffering; it offers psychological, social, and spiritual support for pediatric patients with life-threatening conditions and their families [9, 13, 14]. Pediatricians play an important role in PPC as co-decision-makers and are crucial in initiating PPC [15]. Still, some physicians perceive the PPC decision-making ethically challenging [16, 17].

Since 1970s, PPC has been developed in many countries, most of which are geographically located in high-income western countries [18]. In Belgium, PPC has been integrated into the mainstream health services and has been practiced for more than 30 years [19]. Belgium has established a relatively advanced PPC system through five pediatric liaison teams (PLTs) affiliated to university hospitals, which aim to ensure continuity of care through all care settings for the child with life-threatening conditions [19, 20]. Belgian pediatricians are supported by PPC professional networks [19, 21]. In Mainland China, PPC has a shorter tradition. Chinese PPC has been slowly developed since 2010 [22], and so far, it is still in its early stage [23–28]. PPC there is almost exclusively implemented in hospital-based oncology wards [27]. Available literature indicates that Chinese pediatricians do not receive much professional support, even though PPC is in high demand [25, 29, 30].

As pediatricians play an important role in PPC, as a first step, it is critical to understand their perspectives^1^ on PPC to improve PPC practices. However, to our knowledge, no studies have investigated Chinese and Belgian pediatricians’ perspectives on PPC. Thus, first determining pediatricians’ perspectives on PPC in a country where PPC is just recently beginning to develop, and then contemporaneously comparing these with those in a country where PPC has been developed for years may help to understand the specific characteristics of PPC practices in these countries. Moreover, understanding which factors influence pediatricians’ perspectives on PPC can help stakeholders in China to improve PPC practices. Therefore, the twofold aim of this study was (1) to determine and compare Chinese and Belgian pediatricians’ perspectives on PPC, and (2) to identify factors that influence their perspectives.

## Methods

### Design

We conducted a cross-sectional online survey using the Pediatric Palliative Care Attitude Scale (PPCAS) to investigate pediatricians’ perspectives toward PPC. The Checklist for Reporting of Survey Studies (CROSS) was used as a guide to carry out and report the results of the study [31].

### Participants and sampling

We recruited pediatricians who met the following inclusion criteria: (1) currently practicing in Mainland China or Flanders, Belgium; and (2) capable of reading Chinese or English. After conducting a power analysis using G*Power software (effect size d = 0.5; error probability α = 1%; power = 0.9; allocation ratio = 1.5), we estimated that we would need a minimum sample size of 151 pediatricians in Mainland China and 101 pediatricians in Flanders [32, 33].

### Recruitment

We recruited participants from November 2022 to March 2023, using convenience sampling and snowballing. In Mainland China, first, we emailed pediatrician and PPC associations to ask for helping administer our survey to Chinese members (*n* = 48). Second, we collected pediatricians’ public contact information via institutional websites or professional publications. We contacted individual pediatricians (*n* = 286) in 24 regions via their publicly available email or, more commonly, through WeChat, an instant messaging app ubiquitously used in Mainland China, because email is not as common communication platform there [34, 35]. Third, through WeChat, we also contacted other healthcare providers, e.g., physicians working in other departments and pediatric nurses (*n* = 94), to share our survey to their pediatrician colleagues. In Flanders, first, we emailed the presidents of the Belgian and the Flemish Association for Pediatrics to distribute our survey to Flemish members. Second, we emailed all heads of pediatric departments of Flemish hospitals (*n* = 49) to distribute the survey to their pediatricians. Third, we sent survey invitations to individual pediatricians using their publicly available institutional email (*n* = 423). Finally, we sent a reminder invitation to all individual pediatricians. In both regions, we asked participants to share our invitation with potentially interested pediatrician colleagues. The questionnaire could only be submitted once by the same IP address.

### Data collection

To better understand pediatricians’ perspectives toward PPC, we used the PPCAS, which was language-adapted from the Neonatal Palliative Care Attitude Scale (NiPCAS). The original language for the NiPCAS was English; it contains seven demographic questions and 26 items measuring clinicians’ perspectives on neonatal palliative care [36]. YZ developed and used a culturally and language-adapted version of the NiPCAS translated into Simplified Chinese, the standard language character set used in Mainland China [34]. This Chinese version of the NiPCAS was also evaluated psychometrically [37]. The psychometric information about the original English NiPCAS and the Simplified Chinese NiPCAS are presented in Supplementary Material 1.

To create the PPCAS, we made minor changes to the 26 items of the NiPCAS [33, 34]. For example, we replaced “baby/babies” with “child/children,” “neonatal” with “pediatric,” “neonatal nursing education” with “pediatric education,” and “neonatal nursing” with “pediatric medicine.” The English and the Simplified Chinese versions of the PPCAS questionnaire both consist of two categories of questions: (1) 12 demographic questions (e.g., age, PPC educational experience); and (2) 26 PPC-related questions (e.g., PPC support from work settings, PPC importance). All scale items were closed-ended questions graded on a 5-point Likert response scale, ranging from “0 points” for strongly disagree to “5 points” for strongly agree. We created the English online Survey using Qualtrics, the Chinese one using WJX, and collected data through these two platforms. The two questionnaires are presented in Supplementary Material 2.

### Exploratory factor analysis

The overall Cronbach’s α for the 26-item PPCAS was 0.83, indicating a good general reliability of the scale. We carried out exploratory factor analysis (EFA) to determine the construct of the PPCAS and tested the scale reliability with the new population of pediatricians. We factor analyzed the 26 PPCAS items using principle components method for extraction, and varimax for rotation. The Kaiser-Meyer-Oklin value was 0.86 (over recommended value of 0.60); the Bartlett’s Test of Sphericity was 0.000, indicating that the data were suitable for conducting meaningful EFA.

The EFA produced a five-factor model. However, we removed one factor (comprising items 4, 8, 24, and 25), because its Cronbach’s α was 0.36, indicating that the factor was unreliable (i.e., was not a theoretically meaningful factor). We therefore used a four-factor model that retained 22 of the 26 items. This model explained 52.10% of the total variance. Item loadings are presented in Supplementary Material 3.

The four factors were identified based on the thematic content of items within the factor loadings and group discussions. Items related to support from the work environment, e.g., having supportive policies/guidelines, being allowed to express opinions, having adequate time to implement PPC, were named as the factor Unit Support with a Cronbach’s α of 0.87. Items referred to pediatricians’ internal obstacles of caring for children with life-threatening condition, e.g., feeling uncomfortable or traumatized when a child dies, were named as Personal Obstacles with a Cronbach’s α of 0.71. The items about the importance of PPC, e.g., the importance of PPC in pediatric settings and pediatric education, were named as PPC Importance with a Cronbach’s α of 0.55. And items about palliative care work experience, e.g., experiences of receiving in-service education and providing PPC, were named as Work Experience with a Cronbach’s α of 0.66. Detailed item divisions are presented in Table 1.

**Table 1:** PPCAS Subscale Comparisons Between Groups

Subscale Item	Scores Mean (SD)			Test Statistics^a^	*p*-value^b^
	Mainland China	Flanders	All Participants		
**Unit Support (α = 0.87)** ^**c**^	2.80 (0.89)	3.42 (0.86)	3.02 (0.89)	t=-5.882	**< 0.001**
5. The medical staff supports palliative care for dying children in my work setting	3.69 (1.07)	4.06 (1.25)	3.79 (1.13)		
6. The physical environment of my work setting is ideal for providing palliative care to dying children	2.91 (1.18)	2.65 (1.33)	2.84 (1.23)		
7. My work setting is adequately staffed for providing the needs of dying children requiring palliative care and their families	2.73 (1.20)	2.45 (1.52)	2.66 (1.30)		
13. When a child dies in my work setting, I have sufficient time to spend with the family	2.29 (1.11)	3.35 (1.34)	2.57 (1.26)		
14. There are policies/guidelines to assist in the delivery of palliative care in my work setting	2.36 (1.11)	2.82 (1.40)	2.48 (1.21)		
15. In my work setting, when a diagnosis with a likely poor outcome is made, parents are informed of palliative care options	2.85 (1.21)	3.88 (1.16)	3.12 (1.28)		
16. In my work setting, the team expresses its opinions, values, and beliefs about providing care to dying children	2.77 (1.23)	3.99 (1.20)	3.09 (1.34)		
19. All members of the healthcare team in my work setting agree with and support palliative care when it is implemented for a dying child	3.44 (1.04)	4.19 (1.03)	3.63 (1.09)		
**Personal Obstacles (α = 0.71)**	2.25 (0.58)	3.50 (0.76)	2.58 (0.84)	t=-16.113	**< 0.001**
3. I feel a sense of personal failure when a child dies^d^	1.68 (0.89)	3.56 (1.23)	2.17 (1.29)		
17. Caring for dying children is traumatic for me^d^	2.64 (1.16)	3.47 (1.27)	2.85 (1.24)		
20. In my work setting, the staff go beyond what they feel comfortable with in using technological life support^d^	2.43 (1.16)	3.60 (1.15)	2.74 (1.19)		
21. In my work setting, staff are asked by parents to continue life-extending care beyond what they feel is right^d^	1.84 (0.81)	3.21 (1.31)	2.20 (1.14)		
22. My personal attitude about death affects my willingness to deliver palliative care^d^	2.66 (0.81)	3.68 (1.47)	2.93 (1.30)		
**PPC Importance (α = 0.55)**	3.87 (0.52)	4.51 (0.46)	4.04 (0.57)	t=-11.775	**< 0.001**
1. Palliative care is as important as curative care in the pediatric environment	4.18 (0.94)	4.70 (0.79)	4.32 (0.94)		
10. When children are dying in my work setting, providing pain relief is a priority for me	4.12 (0.91)	4.83 (0.52)	4.30 (0.88)		
12. Palliative care is necessary in pediatric education	4.46 (0.64)	4.73 (0.60)	4.53 (0.64)		
23. Palliative care is against the values of pediatric medicine^d^	3.96 (0.81)	4.78 (0.77)	4.18 (0.99)		
26. Curative care is more important than palliative care in the pediatric intensive care environment^d^	2.62 (1.12)	3.50 (1.38)	2.85 (1.25)		
**Work Experience (α = 0.66)**	2.76 (0.92)	2.92 (0.94)	2.80 (0.93)	t=-1.534	0.126
2. I have had experience of providing palliative care to dying children and their families	2.91 (1.35)	3.62 (1.45)	3.09 (1.41)		
9. My previous experiences of providing palliative care to dying children have been rewarding	2.85 (1.14)	3.84 (1.08)	3.11 (1.21)		
11. I am often exposed to death in the pediatric environment	2.70 (1.35)	2.12 (1.48)	2.55 (1.41)		
18. I have received in-service education that assists me to support and communicate with parents of dying children	2.58 (1.22)	2.08 (1.33)	2.45 (1.27)		

Abbreviation: SD = standard deviation

^a^t: Independent t-test

^b^*p*-value: Comparison between Mainland China mean score and Flanders mean score; <0.05 was considered statistically significant

^c^α: Cronbach’s α value

^d^Indicates items whose scores were coded in SPSS with the opposite valence to how they appear in the PPCAS. Higher scores suggested participants agree in a lesser extent and vice versa. Scores > 3 indicated participants strongly/somewhat disagree; scores < 3 indicated participants strongly/somewhat agree

### Data analysis

We only analyzed fully completed questionnaires. Statistical analyses were performed using IBM SPSS Statistics 28 for Windows. We coded the responses of seven of the test items (items 3, 17, 20, 21, 22, 23, 26) in their opposite-valence form before running the statistics, because of the opposite questioning way. Thus, after recoding, all items had the same valence. Descriptive statistics were used to describe socio-demographic characteristics and item responses. Partial correlation analyses were used to explore potential associations between subscales. In addition, we conducted univariate and multivariate analyses to explore potential factors that might influence pediatricians’ perspectives.

Before determining which statistical tests to use, we assessed whether the data were distributed normally using frequency histograms. For normally distributed data, we used a parametric test (independent t-test) to conduct two-group comparisons (univariate analyses). For non-normally distributed data, we used a non-parametric test (Mann-Whitney U test). Additionally, Spearman’s correlation was used to explore possible associations between socio-demographic characteristics and pediatrician perspectives. For multivariate analyses, we used multiple linear regression, in which we assigned four subscales of the questionnaire as dependent variables, and 12 of the demographic characteristics were assigned as independent variables. Multicollinearity was assessed with collinearity tolerance, variance inflation factor, eigenvalue, condition index, and variance proportions. The statistical analyses we did were evaluated and guided by two biostatisticians having expertise in medical studies. Statistical significance was set at a two-sided *p-value* of < 0.05.

## Results

### Sample characteristics

We received 466 responses: 328 from Mainland China and 138 from Flanders. We excluded three questionnaires from Mainland China because they did not meet our inclusion criteria (currently practicing as pediatricians), and 23 questionnaires from Flanders because they were incomplete. Therefore, we included 440 questionnaires (Mainland China *n* = 325, Flanders *n* = 115) with an overall completion rate of 94.4%, (440/466), 99.1% in Mainland China (325/328), and 83.3% in Flanders (115/138). Over half (53.6%, 236/440) of the participants specialized in general pediatrics; 94.3% (415/440) primarily worked full-time; and 96.4% (424/440) worked on direct patient care. Most participants (88.2%, 388/440) indicated that they had never received PPC education.

The proportion of female-to-male participants varied in the two countries’ groups of participants. The group from Flanders had a higher proportion of females than the group from Mainland China (77.4% vs. 65.5%, *p* = 0.019). Flemish participants were older (44.5 years vs. 35.0 years, *p* < 0.001) and had more years of pediatric experience (14.4 vs. 9.6, *p* < 0.001). Most Chinese participants stated that they had no religious beliefs (90.5% vs. 44.3%, *p* < 0.001). More of them worked in university hospitals than their Flemish counterparts (52.3% vs. 38.3%, *p* = 0.010). On the other hand, more Flemish participants had experience caring for children with life-threatening condition (70.4% vs. 36.3%, *p* < 0.001) and providing PPC (55.7% vs. 12.0%, *p* < 0.001) compared to Chinese participants. Detailed socio-demographic characteristics of the participants are presented in Table 2.

**Table 2:** Socio-demographic Characteristics of Study Participants (*n* = 440)

Variable		Mainland China *n* = 325 (73.9%)	Flanders *n* = 115 (26.1%)	Total *n* = 440 (100%)	Test Statistic	*p*-value^a^
Gender	Female, n (%)	213 (65.5)	89 (77.4)	30 (68.6)	χ²=5.544	**0.019**
	Male, n (%)	112 (34.5)	26 (22.6)	13 (31.4)		
Age	mean (SD)	35.0 (7.8)	44.5 (9.8)	37.5 (9.3)	t=-9.409	**< 0.001**
Religious beliefs	No religion, n (%)	294 (90.5)	51 (44.3)	345 (78.4)	χ²=106.697	**< 0.001**
	Have religious beliefs, n (%)	31 (9.5)	64 (55.7)	95 (21.6)		
Professional specialty	General pediatrician, n (%)	171 (51.6)	65 (56.5)	236 (53.6)	χ²=0.521	0.470
	Pediatric specialist, n (%)	154 (48.4)	50 (43.5)	204 (46.4)		
Institutional setting	University hospital, n (%)	170 (52.3)	44 (38.3)	214 (48.6)	χ²=6.709	**0.010**
	Regional hospital and other setting, n (%)	155 (47.7)	71 (61.7)	226 (51.4)		
Work department	General pediatric ward, and private practice space n (%)	255 (78.5)	99 (86.1)	354 (80.5)	χ²=3.141	0.076
	Pediatric subspecialty ward, n (%)	70 (21.5)	16 (13.9)	86 (19.5)		
Main work	Direct patient care, n (%)	309 (95.1)	115 (100)	424 (96.4)	χ²=4.554	**0.033**
	Medical management, research, or education, n (%)	16 (4.9)	0 (0)	16 (3.6)		
Employment status	Full-time, n (%)	319 (98.2)	96 (83.5)	415 (94.3)	χ²=34.138	**< 0.001**
	Part-time, n (%)	6 (1.8)	19 (16.5)	25 (5.7)		
Years of being pediatrician	mean (SD)	9.6 (8.1)	14.4 (9.6)	10.8 (8.8)	t=-4.836	**< 0.001**
Received PPC Education	Yes, n (%)	38 (11.7)	14 (12.2)	52 (11.8)	χ²=0.019	0.891
	No, n (%)	287 (88.3)	101(87.8)	388 (88.2)		
Experience caring for dying children	Yes, n (%)	118 (36.3)	81 (70.4)	199 (45.2)	χ²=39.936	**< 0.001**
	No, n (%)	207 (63.7)	34 (29.6)	241 (54.8)		
Experience providing PPC	Yes, n (%)	39 (12.0)	64 (55.7)	103 (23.4)	χ²=90.277	**< 0.001**
	No, n (%)	286 (88.0)	51 (44.3)	337 (76.6)		

Abbreviations: PPC = pediatric palliative care; SD = standard deviation; χ²=chi-square test; t = independent t-test

^a^*p*-value: Comparison between Mainland China participants and Flanders participants; <0.05 was considered statistically significant

### Perspectives of Chinese and Flemish pediatricians toward PPC

Factors identified in the EFA guided how we reported (below) pediatricians’ perspectives toward PPC (see Tables 1 and 3).

**Table 3:** Distribution of Responses on the PPCAS

PPCAS item (α = 0.83)	Mainland China (*n* = 325)			Flanders (*n* = 115)			All (*n* = 440)		
	Strongly/ Somewhat Disagree, n (%)	Strongly/ Somewhat Agree, n (%)	Unsure, n (%)	Strongly/ Somewhat Disagree, n (%)	Strongly/ Somewhat Agree, n (%)	Unsure, n (%)	Strongly/ Somewhat Disagree, n (%)	Strongly/ Somewhat Agree, n (%)	Unsure, n (%)
1. Palliative care is as important as curative care in the pediatric environment	21 (6.5)	277 (85.2)	27 (8.3)	6 (5.2)	109 (94.8)	0 (0)	27 (6.1)	386 (87.7)	27 (6.1)
2. I have had experience of providing palliative care to dying children and their families	136 (41.8)	125 (38.5)	64 (19.7)	35 (30.4)	79 (68.7)	1 (0.9)	171 (38.9)	204 (46.4)	65 (14.8)
3. I feel a sense of personal failure when a child dies	27 (8.3)	288 (88.7)	10 (3.1)	74 (64.3)	38 (33.0)	3 (2.6)	101 (23.0)	326 (74.1)	13 (3.0)
4. There is support for pediatric palliative care in society	30 (9.2)	263 (80.9)	32 (9.8)	30 (26.1)	80 (69.6)	5 (4.3)	60 (13.6)	343 (78.0)	37 (8.4)
5. The medical staff supports palliative care for dying children in my work setting	52 (16.0)	215 (66.2)	58 (17.8)	19 (16.5)	89 (77.4)	7 (6.1)	71 (16.1)	304 (69.1)	65 (14.8)
6. The physical environment of my work setting is ideal for providing palliative care to dying children	142 (43.7)	113 (34.8)	70 (21.5)	69 (60.0)	45 (39.1)	1 (0.9)	211 (48.0)	158 (35.9)	71 (16.1)
7. My work setting is adequately staffed for providing the needs of dying children requiring palliative care and their families	170 (52.3)	102 (31.4)	53 (16.3)	74 (64.3)	40 (34.8)	1 (0.9)	244 (55.5)	142 (32.3)	54 (12.3)
8. In my work setting, parents are involved in decisions about their dying child	53 (16.3)	226 (69.5)	46 (14.2)	7 (6.1)	94 (81.7)	14 (12.2)	60 (13.6)	320 (72.7)	60 (13.6)
9. My previous experiences of providing palliative care to dying children have been rewarding	132 (40.6)	103 (31.7)	90 (27.7)	14 (12.2)	81 (70.4)	20 (17.4)	146 (33.2)	184 (41.8)	110 (25.0)
10. When children are dying in my work setting, providing pain relief is a priority for me	132 (40.6)	103 (31.7)	90 (27.7)	0 (0)	108 (93.9)	7 (6.1)	132 (30.0)	211 (48.0)	97 (22.0)
11. I am often exposed to death in the pediatric environment	192 (59.1)	122 (37.5)	11 (3.4)	86 (74.8)	29 (25.2)	0 (0)	278 (63.2)	151 (34.3)	11 (2.5)
12. Palliative care is necessary in pediatric education	2 (0.6)	306 (94.2)	17 (5.2)	1 (0.9)	108 (93.9)	6 (5.2)	3 (0.7)	414 (94.1)	23 (5.2)
13. When a child dies in my work setting, I have sufficient time to spend with the family	224 (68.9)	67 (20.6)	34 (10.5)	42 (36.5)	67 (58.3)	6 (5.2)	266 (60.4)	134 (30.4)	40 (9.1)
14. There are policies/guidelines to assist in the delivery of palliative care in my work setting	202 (62.2)	58 (17.8)	65 (20.0)	57 (49.6)	50 (43.4)	8 (7.0)	259 (58.9)	108 (24.5)	73 (16.6)
15. In my work setting, when a diagnosis with a likely poor outcome is made, parents are informed of palliative care options	154 (47.4)	119 (36.6)	52 (16.0)	18 (15.7)	82 (71.3)	15 (13.0)	172 (39.1)	201 (45.7)	67 (15.2)
16. In my work setting, the team expresses its opinions, values, and beliefs about providing care to dying children	153 (47.1)	109 (33.5)	63 (19.4)	17 (14.8)	88 (76.5)	10 (8.7)	170 (38.6)	197 (44.8)	73 (16.6)
17. Caring for dying children is traumatic for me	97 (29.8)	183 (56.3)	45 (13.8)	70 (60.9)	41 (35.7)	4 (3.5)	167 (38.0)	224 (50.9)	49 (11.1)
18. I have received in-service education that assists me to support and communicate with parents of dying children	184 (56.6)	93 (28.6)	48 (14.8)	85 (73.9)	29 (25.2)	1 (0.9)	269 (61.1)	122 (27.7)	49 (11.1)
19. All members of the healthcare team in my work setting agree with and support palliative care when it is implemented for a dying child	184 (56.6)	93 (28.6)	48 (14.8)	9 (7.8)	91 (79.1)	15 (13.0)	193 (43.9)	184 (41.8)	63 (14.3)
20. In my work setting, the staff go beyond what they feel comfortable with in using technological life support	72 (22.2)	209 (64.3)	44 (13.5)	72 (62.6)	22 (19.1)	21 (18.3)	144 (32.7)	231 (52.5)	65 (14.8)
21. In my work setting, staff are asked by parents to continue life-extending care beyond what they feel is right	20 (6.2)	289 (88.9)	16 (4.9)	53 (46.1)	44 (38.3)	18 (15.7)	73 (16.6)	333 (75.7)	34 (7.7)
22. My personal attitude about death affects my willingness to deliver palliative care	94 (28.9)	187 (57.5)	44 (13.5)	75 (65.2)	33 (28.7)	7 (6.1)	169 (38.4)	220 (50.0)	51 (11.6)
23. Palliative care is against the values of pediatric medicine	252 (77.5)	35 (10.8)	38 (11.7)	111 (96.5)	4 (3.5)	0 (0)	363 (82.5)	39 (8.9)	38 (8.6)
24. When a child dies in my work setting, counselling is available if I need it	45 (13.8)	245 (75.4)	35 (10.8)	24 (20.9)	73 (63.5)	18 (15.7)	69 (15.7)	318 (72.3)	53 (12.0)
25. There is a belief in society that children should not die, under any circumstances	166 (51.1)	129 (39.7)	30 (9.2)	59 (51.3)	53 (46.1)	3 (2.6)	225 (51.1)	182 (41.4)	33 (7.5)
26. Curative care is more important than palliative care in the pediatric intensive care environment	107 (32.9)	185 (56.9)	33 (10.2)	71 (61.7)	37 (32.2)	7 (6.1)	178 (40.5)	222 (50.5)	40 (9.1)

Abbreviations: PPCAS = Pediatric Palliative Care Attitude Scale; α = Cronbach’s α value

### *Unit support*

Pediatricians agreed that their work settings supported PPC implementations (mean ± SD 3.02 ± 0.89), especially for Flemish pediatricians (Flemish [F]: 3.42 ± 0.86; Chinese [C]: 2.80 ± 0.89; t=-5.882, *p* < 0.001). Specifically, more Flemish pediatricians reported having sufficient time to spend with the family after their child died (F: 3.35 ± 1.34; C: 2.29 ± 1.11); were allowed to express their opinions, values, and beliefs about PPC (F: 3.99 ± 1.20; C: 2.77 ± 1.23); and that parents were informed about the PPC options for a child diagnosed with potentially poor outcome (F: 3.88 ± 1.16; C: 2.85 ± 1.21).

In both regions, however, most of the pediatricians disagreed having ideal physical environment (F: 2.65 ± 1.33; C: 2.91 ± 1.18), adequate staff (F: 2.45 ± 1.52; C: 2.73 ± 1.20), or supportive policies/guidelines to assist the PPC delivery (F: 2.82 ± 1.40; C: 2.36 ± 1.11).

### *Personal obstacles*

Pediatricians faced internal obstacles specific to them while caring for children with life-threatening condition (2.58 ± 0.84), especially for Chinese pediatricians (F: 3.50 ± 0.76; C: 2.25 ± 0.58; t=-16.113, *p* < 0.001). Particularly, Chinese pediatricians reported more frequently two factors as personal obstacles: feelings of personal failure when a child dies (F: 3.56 ± 1.23; C:1.68 ± 0.89) and dealing with parents’ requests to continue life-extending care beyond what they feel is right (F: 3.21 ± 1.31; C: 1.84 ± 0.81). Moreover, they felt more traumatized while caring for a child with life-threatening condition (F: 3.47 ± 1.27; C: 2.64 ± 1.16) and more uncomfortable using technological life support (F: 3.60 ± 1.15; C: 2.43 ± 1.16) than their Flemish counterparts. Their willingness to provide PPC also appeared to be influenced more by their personal beliefs about death (F: 3.68 ± 1.47; C: 2.66 ± 0.81).

### *Pediatric palliative care importance*

Most pediatricians considered PPC important (4.04 ± 0.57). In both regions, pediatricians considered PPC as important as curative treatment in the pediatric environment (F: 4.70 ± 0.79; C: 4.18 ± 0.94); PPC to be necessary in pediatric education (F: 4.73 ± 0.60; C: 4.46 ± 0.64); and providing pain relief to be a priority when caring for a child with life-threatening condition (F: 4.83 ± 0.52; C: 4.12 ± 0.91). Chinese pediatricians, however, had a lower mean score on this subscale (F: 4.51 ± 0.46; C: 3.87 ± 0.52; t=-11.775, *p* < 0.001). Specifically, Chinese pediatricians considered curative treatment care being more important than PPC in the pediatric intensive care units (PICUs) (F: 3.50 ± 1.38; C: 2.62 ± 1.12).

### *Work experience*

Pediatricians have limited PPC experience (2.80 ± 0.93). More Flemish pediatricians provided PPC to a child with life-threatening condition and their family (F: 3.62 ± 1.45; C: 2.91 ± 1.35) and considered their PPC experiences to be rewarding (F: 3.84 ± 1.08; C: 2.85 ± 1.14). However, in both regions, pediatricians did not agree that they had received additional training to help support or communicate with parents of a child with life-threatening condition (F: 2.08 ± 1.33; C: 2.58 ± 1.22); nor that they were frequently exposed to death in the pediatric environment (F: 2.12 ± 1.48; C: 2.70 ± 1.35).

### Associations between Chinese and Flemish pediatricians’ perspectives and socio-demographic characteristics

We conducted univariate and multivariate analyses to determine which factors, if any, influenced pediatricians’ perspectives toward PPC. Tables 4 and 5 present the results of the multivariate analyses for Mainland China and Flanders, respectively. The results of the univariate analysis are presented in Supplementary Materials 4 and 5.

**Table 4:** Multiple Linear Regression Analysis of Mainland Chinese Pediatricians’ Socio-characteristics and Perspectives Toward PPC^a^

Variable	Variable Category	Estimate	*p*-value^d^	95%CI
**Unit Support** ^b^				
Constant	NA	2.737	< 0.001	(2.644, 2.830)
Received PPC education	Yes	0.850	< 0.001	(0.561, 1.140)
	No	Ref	NA	NA
Experience providing PPC	Yes	0.358	0.014	(0.072, 0.644)
	No	Ref	NA	NA
**PPC Importance** ^b^				
Constant	NA	3.280	< 0.001	(3.022, 3.538)
Age	Per unit increase	0.013	< 0.001	(0.006, 0.020)
Gender	Female	0.134	0.022	(0.019, 0.249)
	Male	Ref	NA	NA
Experience caring for dying children	Yes	0.124	0.032	(0.011, 0.238)
	No	Ref	NA	NA
**Work Experience** ^**b**^				
Constant	NA	3.082	< 0.001	(2.869, 3.296)
Work department^c^	General pediatric Ward, and private practice space	-0.410	< 0.001	(-0.651, -0.168)
	Pediatric subspecialty ward	Ref	NA	NA

Abbreviations: PPC = pediatric palliative care; NA = not available; Estimate = unstandardized coefficient B estimate; Ref = reference category; 95% CI = 95% confidence interval

^a^The following independent variables were included in our multiple linear regression model for all subscales except for the Personal Obstacles subscale: gender, age, religious beliefs, professional specialty, institutional setting, work department, main work, employment status, years of being pediatrician, received PPC education, experience caring for dying children, and experience providing PPC. For Personal Obstacles, no independent variables were included

^b^The following dependent variables were included in our multiple linear regression model for the mean scores of the following subscales: Unit Support (Model: *R* = 0.397, R^2^ = 0.157, *p* < 0.001); PPC Importance (Model: *R* = 0.270, R^2^ = 0.073, *p* < 0.001); and Work Experience (Model: *R* = 0.183, R^2^ = 0.033, *p* < 0.001)

^c^Three independent variables were excluded from analysis: received PPC education, experience caring for dying children, and experience providing PPC. They were excluded because they were largely correlated with the items in the Work Experience subscale

^d^*p*-value: <0.05 was considered statistically significant

**Table 5:** Multiple Linear Regression on Flemish Pediatricians’ Socio-characteristics and Perspectives Toward PPC^a^

Variable	Variable Category	Estimate	*p*-value^d^	95%CI
**Unit Support** ^b^				
Constant	NA	2.862	< 0.001	(2.673, 3.052)
Institutional setting	University hospital	0.911	< 0.001	(0.648, 1.174)
	Regional hospital and other setting	Ref	NA	NA
Experience providing PPC	Yes	0.452	< 0.001	(0.197, 0.707)
	No	Ref	NA	NA
**PPC Importance** ^**b**^				
Constant	NA	4.371	< 0.001	(4.218, 4.523)
Experience caring for dying children	Yes	0.197	0.034	(0.015, 0.379)
	No	Ref	NA	NA
**Work Experience** ^**b**^				
Constant	NA	3.040	< 0.001	(2.703, 3,377)
Professional^c^ specialty	General pediatrician	-0.622	< 0.001	(-0.979, -0.265)
	Pediatric specialist	Ref	NA	NA
Institutional setting	University hospital	0.593	0.002	(0.229, 0.957)
	Regional hospital and other setting	Ref	NA	NA

Abbreviations: PPC = pediatric palliative care; NA = not available; Estimate = unstandardized coefficient B estimate; Ref = reference category; 95% CI = 95% confidence interval

^a^ The following independent variables were included in our multiple linear regression model for all subscales except for the Personal Obstacles subscale: gender, age, religious beliefs, professional specialty, institutional setting, work department, employment status, years of being pediatrician, received PPC education, experience caring for dying children, and experience providing PPC. For Personal Obstacles, no independent variables were included

^b^ The following dependent variables were included in our multiple linear regression model for the mean scores of the following subscales: Unit Support (Model: *R* = 0.650, R^2^ = 0.423, *p* < 0.001); PPC Importance (Model: *R* = 0.198, R^2^ = 0.039, *p* = 0.034); and Work Experience (Model: *R* = 0.564, R^2^ = 0.318, *p* < 0.001)

^c^ Three independent variables were excluded from analysis: received PPC education, experience caring for dying children, and experience providing PPC. They were excluded because they were largely correlated with items in the Work Experience subscale

^d^*p*-value: <0.05 was considered statistically significant

The two groups differed regarding which socio-demographics characteristics influenced their perspectives toward PPC. For Mainland China, those pediatricians who had received PPC education (unstandardized coefficient B estimate [Estimate]: 0.850, 95% confidence interval [CI]: 0.561–1.140, *p* < 0.001) and who had experience in providing PPC (Estimate: 0.358, 95% CI: 0.072–0.644, *p* = 0.014) were more likely to agree that their work settings were supportive of PPC implementations. Additionally, female pediatricians (Estimate: 0.134, 95% CI: 0.019–0.249, *p* < 0.022); older pediatricians (Estimate: 0.013, 95% CI: 0.006–0.020, *p* < 0.001); and those who had experiences in caring for children with life-threatening condition (Estimate: 0.124, 95% CI: 0.011–0.238, *p* < 0.032) were more likely to consider PPC important. Furthermore, pediatricians working in pediatric subspecialty wards (Estimate: -0.410, 95% CI: -0.651- -0.168, *p* < 0.001) were more likely to agree that they had PPC experience.

For Flanders, pediatricians working in university hospitals (Estimate: 0.911, 95% CI: 0.511–1.085, *p* < 0.001) and those who had experience in providing PPC (Estimate: 0.452, 95% CI: 0.648–1.174, *p* < 0.001) were more likely to agree that their work settings were supportive. Pediatricians who had experiences of caring for children with life-threatening condition there (Estimate: 0.197, 95% CI: 0.015–0.379, *p* = 0.034) were more likely to consider PPC important. Furthermore, pediatric subspecialists (Estimate: -0.622, 95% CI: -0.979 - -0.265, *p* < 0.001) and pediatricians working in university hospitals (Estimate: 0.593, 95% CI: 0.229–0.957, *p* = 0.002) were more likely to agree that they had PPC experience.

### Correlations between unit support, personal obstacles, PPC Importance, and work experience

We conducted partial correlation analyses to determine whether the pediatricians’ scores on the four subscales were correlated (Table 6). In both regions, pediatricians’ PPC experience was positively and moderately correlated with the perceived support provided by their work units (ρ_F_ = 0.454; ρ_C_ = 0.661). Additionally, Flemish pediatricians’ perspectives toward the importance of PPC were positively but weakly correlated with internal obstacles they faced (ρ_F_ = 0.230).

**Table 6:** Correlations between Unit Support, Personal Obstacles, PPC Importance, and Work Experience^a^

Chinese					Flemish				
Variable	Unit Support	Personal Obstacles	PPC Importance	Work Experience	Variable	Unit Support	Personal Obstacles	PPC Importance	Work Experience
**Unit Support**	-	**-**	**-**	**-**		-	-	-	**-**
**Personal Obstacles**	0.002^b^	**-**	**-**	**-**		-0.142	-	-	**-**
**PPC Importance**	0.073	0.001	**-**	**-**		0.166	0.230^c^	-	**-**
**Work Experience**	0.661^d^	-0.104	-0.048	**-**		0.454^d^	0.086	0.175	**-**

^a^Partial correlation analysis: When assessing correlations between two variables, the two remaining variables were set as control variables

^b^Partial correlation coefficients ρ: >0.7 highly correlated,; >0.4 and < 0.7 moderately correlated; <0.4 weakly correlated

^c^*p*-value < 0.05

^d^*p*-value < 0.01

## Discussion

Our analyses of PPCAS responses revealed some similarities between Chinese and Flemish pediatricians’ perspectives toward PPC. There were also some important differences that have practical implications. Although both Chinese and Flemish pediatricians do not have extensive PPC experience, Chinese pediatricians perceived less unit support at their place of practice, considered PPC less important than other kinds of care, especially in PICUs, and faced more internal obstacles while practicing PPC. In both regions, pediatricians’ PPC experience and their perceived unit support were correlated. These two factors played an important role in influencing pediatricians’ perspectives toward PPC.

### The importance of PPC Experience

Pediatricians’ PPC experience is an important factor that influences their perspectives toward PPC. In multivariate analyses, we found that in both regions, pediatricians who have experience providing PPC were more likely to perceive their work settings supportive. Two previous qualitative studies confirmed the importance of PPC experience, reporting that improving PPC experience is the best way to improve PPC implementation [38]. This could help to integrate PPC into pediatric oncology care [39]. Also, the Royal College of Paediatrics and Child Health in the UK concluded that previous experiences in PPC-related work help pediatricians make PPC decisions for children with life-threatening condition and their family [40].

However, in this study, pediatricians in both regions have limited PPC education, limited exposure to death, and limited PPC practice. The reasons for this observation might differ in Flanders, Belgium and Mainland China. In Belgium, PPC is offered through five specialized PLTs attached to university hospitals [19, 41, 42]. For most children, PPC is delivered in the family’s home by these specialized teams [19]. Therefore, few of our Flemish participants have extensive PPC experience in comparison with those pediatricians working in the PLTs. Our multivariate analyses confirmed this observation, showing that Flemish pediatric specialists working in university hospitals were more likely to have had PPC experience.

In Mainland China, most of those who do practice PPC, work primarily in the eastern, more developed regions of China [25–28]. In our study, although the pediatricians were recruited from all parts of Mainland China, most work in general pediatric wards and thus had limited PPC experience compared with those working in pediatric subspecialty wards. Therefore, Cai et al. (2021) strongly recommended incorporating PPC into all healthcare systems, including in those less developed regions so that more pediatricians can practice it [28].

### Unit support as a driving force for PPC implementation

Our partial correlation tests showed that PPC experience was correlated with supportive work settings. Hence, improving unit support where pediatricians work could facilitate PPC implementations. A previous Chinese survey study found that healthcare providers working in modern, well-equipped facilities having advanced medical resources were more likely to have opportunities to practice PPC [30]. Additionally, two other qualitative studies stressed the importance of unit support on PPC implementation. In UK, well-functioning and collaborative teams were identified as an enabling factor and as a rewarding aspect of work that made healthcare providers more comfortable to implement PPC [43]. A Canadian study identified supportive work settings as a PPC communication enhancer, because they ensured that the location where PPC occurred was comfortable and appropriate, that sufficient time for PPC was budgeted, and that the family felt supported [38].

In our study, fewer Chinese pediatricians reported having supportive work settings compared with their Flemish counterparts. Mainland China has only one professional PPC association, the PPC subspecialty group of the Pediatrics Society of the Chinese Medical Association [28]. Few institutions support PPC implementation, and if they do, necessary services and personnel are limited [25, 26, 28]. Indeed, the deficiency in PPC-related resources has been a challenge to PPC implementation in Mainland China [25]. In 2021, Cai et al. emphasized that the facilities in Mainland China are insufficient and cannot meet the growing demand for PPC; thus, they recommended establishing dedicated interdisciplinary PPC teams to help mitigate this shortfall [28]. The five PLTs in Belgium could be a good example, which covered and improved PPC implementation for all pediatric patients.

Specifically, in our study, Chinese pediatricians reported that they lacked supportive policies/guidelines for PPC implementation. This was consistent with Zhu et al. who also reported a lack of supportive PPC policies/guidelines for healthcare providers [30]. A survey study [28] and a secondary data analysis study [25] identified lack of supportive policies/guidelines as the largest barrier to PPC implementation in Mainland China. These studies emphasized the urgent need for clear and supportive PPC policies/guidelines and suggested integrating PPC into the Chinese government’s working blueprint, a plan for future working goals.

Lack of PPC policies and guidelines goes beyond China. Studies from the US [44] and Japan [45–47] found that lack of supportive policies/guidelines hinders PPC decision-making. Conversely, a review confirmed that supportive policies/guidelines is a facilitator of PPC implementation and recommended that supportive documents should be published [48].

### Importance of PPC in PICUs and personal obstacles

We found that in both regions, pediatricians agreed with the importance of PPC in general. However, our analyses revealed that in PICUs, Chinese pediatricians perceived curative treatments more important than PPC compared to their Flemish counterparts. In Mainland China, PPC is not viewed by the general public as a valued care approach for children with life-threatening condition [28]. Furthermore, access to PPC is limited, being mainly available in more developed regions [28]. Therefore, many Chinese pediatricians have poor knowledge about PPC [30]. For instance, they find the definition and goals of PPC to be unclear. This might explain why many Chinese pediatricians prefer to continue curative treatments, especially in high-risk settings like PICUs. To improve PPC education, we recommend developing specific PPC-related training/courses for healthcare providers so to improve PPC-related skills, both physical and mental. These training/courses should not focus solely on the clinical symptoms assessment and management, e.g., pain assessment and management, but should take a more holistic approach that includes improving counselling, and addressing psychological and spiritual needs of the family, e.g., communication skills to break bad news, build up a trustful relationship with the family. These recommendations were also supported by some empirical studies conducted in Mainland China [49, 50].

The perceived importance of curative treatment can also be linked to some internal obstacles. In fact, Chinese pediatricians faced more internal obstacles in the process of PPC implementation compared to Belgian pediatricians. Chinese participants in our study, felt a sense of failure when a child dies. This sentiment is echoed in the 2022 study of Gu et al., who reported that Chinese neonatologists express feelings of failure when caring for dying neonates [51]. Chinese cultural background may also contribute to the pediatricians’ perceived importance of curative treatments in highly specialized settings like PICUs and the internal obstacles they face while practicing PPC. According to traditional Chinese philosophies (e.g., Taoism, Confucianism, and Buddhism), talking about death is “taboo” [51–55]. Thus, pediatricians typically avoid discussing death with patients and their family [51–55]. This is consistent with other studies that found that avoidance of death discussions due to cultural influences was a barrier to PPC implementation [49, 56, 57]. Moreover, in China the lack of “death education” (i.e., formal training about death) exacerbates this view, also making it more difficult to discuss death [58, 59]. In this kind of environment, healthcare providers are assumed to try all possible means to prolong a patient’s life [60, 61]. These are substantial socio-cultural barriers to PPC implementations in Mainland China.

As an essential component of PPC, advance care planning (ACP) has also been hindered by the traditional Chinese philosophies [62]. ACP in pediatrics has been discussed in Mainland China more recently, though it has been adapting to the socio-culture there [62, 63]. For instance, at the beginning of 2024, the first Chinese ACP intervention protocol for adolescents in cancer has been developed for improving ACP practices, but its effectiveness remains to be validated [64]. The Chinese version of “Voicing My CHOiCES” was the first available ACP document for adolescents, helping adolescents to express wishes and preferences, and improve ACP communications [65]. However, it was culturally adapted and applied recently [65]. Moreover, an ACP preparedness investigation found only few Chinese parents were prepared for having ACP for their child [66].

Conversely, Belgium has built up a high-performance ACP system, which is strongly supported by national legislation and policies [20]. The government legislation supports the development and embedding of ACP throughout the whole healthcare system, including Flanders [20]. For instance, the development of a complex intervention program, Benefts of Obtaining Ownership Systematically Together (BOOST) in paediatric Advance Care Planning, was supported by the patients, parents, healthcare providers, and PPC experts in Flanders, and was considered appropriate and feasible [67].

### Strengths and limitations

To our knowledge, this is the first study to use a validated instrument to determine and to compare perspectives toward PPC of pediatricians from a country that is just developing PPC with those from a country with long-established PPC. This comparison highlighted clear similarities and differences in pediatricians’ perspectives. Another strength is the methodological rigor of the study, which was enhanced by the input of two biostatisticians and the use of CROSS.

Some limitations must be acknowledged. First, the PPC Importance subscale has lower reliability (Cronbach’s α of 0.55); thus, the results of this subscale might be less consistent. Second, it might be the case that pediatricians with similar scores might have different perspectives. The nuanced reality behind pediatricians’ perspectives remains somewhat unclear. Qualitative studies are needed to explore pediatricians’ underlying reasons explaining their perspective. Third, our results should not be used to infer causal relationships between pediatricians’ socio-demographic characteristics and their perspectives toward PPC. Fourth, many of the socio-demographic characteristics of the recruited pediatricians in the two regions has significant differences, e.g., age, religious beliefs, PPC work experiences, which might have hindered the precise comparison of pediatricians’ perspectives due to the diversity of the groups. Fifth, our sample size was relatively small; thus, our results cannot be generalized to all pediatricians in Mainland China and Flanders. The sample of pediatricians we recruited, however, is diverse (i.e., different institutional settings), which is an important strength of this study.

## Conclusions

Chinese pediatricians experienced more barriers in practicing PPC than Flemish pediatricians, such as less support from their units. Since PPC experience influences how pediatricians perceive PPC, it is important to provide more opportunities for PPC experiences. Our results may give some insights on PPC improvement for other countries which are supporting PPC implementation.

### Electronic supplementary material

Below is the link to the electronic supplementary material.

Supplementary Material 1

Supplementary Material 2

Supplementary Material 3

Supplementary Material 4

Supplementary Material 5

## Data Availability

Data is provided within the manuscript or supplementary information files.

## Footnotes

1 In this study, “perspectives” referred to attitudes and experiences associated with PPC.

## Abbreviations

PPC: pediatric palliative care
PPCAS: Pediatric Palliative Care Attitude Scale
CROSS: Checklist for Reporting of Survey Studies
NiPCAS: Neonatal Palliative Care Attitude Scale
EFA: exploratory factor analysis
PICUs: pediatric intensive care units
