# Supplementary material for: Chinese and Belgian pediatricians’ perspectives toward pediatric palliative care: an online survey
Source: BMC Palliat Care. 2024 Apr 23;23:106. doi: 10.1186/s12904-024-01436-0 (PMC11036583; doi:10.1186/s12904-024-01436-0)
Supplement: Supplementary file 1 — Supplementary Material 1 [file 12904_2024_1436_MOESM1_ESM.docx]

**Supplementary Material 1. Complementary information for the Neonatal Palliative Care Attitude Scale (NiPCAS)**

| **Original English version of the NiPCAS** |
| --- |
| The original language for the NiPCAS was English; it contains seven demographic questions and 26 items measuring clinicians’ perspectives on neonatal palliative care [1].  Based on exploratory factor analysis, 12 of the 26 items were divided into three subscales: (1) Organization (work environment), (2) Resources (human and material resources), and (3) Clinician (moral and ethical concerns of the clinicians) [1]. These subscales were used to identify barriers to and facilitators of neonatal palliative care practices [1]. The remaining 14 items relate to clinicians’ attitudes and experiences [1]. These were not subdivided into subscales.  The overall Cronbach’s α of the original English scale was 0.77 [2]. The Cronbach’s α values for its three subscales were 0.73 for Organization, 0.65 for Resources, and 0.63 for Clinician [1], which indicates that the scale has acceptable reliability.  The NiPCAS had been translated into Persian [3], Italian [4], Turkish [5, 6], Korean [7], Czech [8], Portuguese [9], Traditional Chinese [10], and Simplified Chinese [11, 12], and has been applied respectively in those contexts with acceptable reliability and validity. |
| **Simplified Chinese version of the NiPCAS** |
| [Anonymous] developed and used a culturally and language-adapted version of the NiPCAS translated into Simplified Chinese, the standard language character set used in Mainland China [12]. This Chinese version of the NiPCAS was also evaluated psychometrically [12].  The Simplified Chinese version of the NiPCAS comprises a total of 26 items, which are divided into five subscales: (1) Organization (work environment), (2) Resources (human and material resources), (3) Work Experience (palliative care related work experience), (4) Beliefs (palliative care related viewpoints), and (5) Barriers (negative factors). These subscales have been used to identify the barriers to and facilitators of neonatal palliative care practices [12].  The overall Cronbach’s α of the Simplified Chinese scale ranged from 0.74 [12] to was 0.87 [11]. The Cronbach’s α values for the subscales were 0.77 for Organization, 0.80 for Resources, 0.60 for Work Experience, 0.63 for Beliefs, and 0.66 for Barriers [12]. |

**References**

[1] Kain V, Gardner G, Yates P. Neonatal palliative care attitude scale: development of an instrument to measure the barriers to and facilitators of palliative care in neonatal nursing. Pediatrics 2009;123(2):e207-13.

[2] Chin SDN, Paraszczuk AM, Eckardt P, Bressler T. Neonatal nurses' perceptions of palliative care in the neonatal intensive care nnit. MCN Am J Matern Child Nurs 2021;46(5):250-257.

[3] Azzizadeh Forouzi M, Banazadeh M, Ahmadi JS, Razban F. Barriers of palliative care in neonatal intensive care units. Am J Hosp Palliat Care 2017;34(3):205-211.

[4] Cerratti F, Tomietto M, Della Pelle C, Kain V, Di Giovanni P, Rasero L, Cicolini G. Italian nurses' attitudes towards neonatal palliative care: a cross-sectional survey. J Nurs Scholarsh 2020;52(6):661-670.

[5] Akay G, Aytekin Özdemir A. Validity and reliability of the Neonatal Palliative Care Attitude Scale in Turkey. Florence Nightingale J Nurs 2021;29(2):212-220.

[6] Erel BN, Büyük ET. The effect of emotional labor levels on the attitudes of neonatal intensive care nurses towards palliative care. JOMPAC 2021;2(2):40-46.

[7] Jung HN, Ju HO. Comparison of the attitudes of nurses and physicians toward palliative care in neonatal intensive care units. J Hosp Palliat Care 2021;24(3):165-173.

[8] Kachlová M, Bužgová R. The attitudes of neonatological nurses to providing perinatal palliative care. Kontakt 2021;23(4):240-246.

[9] Sousa FP, Roldão MG, Rebotim AM, et al. The Neonatal Palliative Care Attitude Scale: psychometric properties for Portuguese neonatal nurses. Palliat Support Care 2022;12:1-6.

[10] Chen CH, Huang LC, Liu HL, et al. To explore the neonatal nurses' beliefs and attitudes towards caring for dying neonates in Taiwan. Matern Child Health J 2013;17(10):1793-801.

[11] Gu L, Li ZZ, Peng NH, et al. Barriers to and facilitators of neonatal palliative care among neonatal professionals in China. Am J Hosp Palliat Care 2022;39(6):695-700.

[12] Zhong Y, Black BP, Kain VJ, Sun X, Song Y. Development of the Simplified Chinese version of Neonatal Palliative Care Attitude Scale. Front Pediatr 2022;10:962420.
