# Supplementary material for: Chinese and Belgian pediatricians’ perspectives toward pediatric palliative care: an online survey
Source: BMC Palliat Care. 2024 Apr 23;23:106. doi: 10.1186/s12904-024-01436-0 (PMC11036583; doi:10.1186/s12904-024-01436-0)
Supplement: Supplementary file 2 — Supplementary Material 2 [file 12904_2024_1436_MOESM2_ESM.docx]

**Supplementary Material 2. The Pediatric Palliative Care Attitude Scale (PPCAS)**

**English Version**

***The Pediatric Palliative Care Attitude Scale***

***(PPCAS)***

**Section One:**

*Firstly, we would like to ask some questions about you. Please specify your answer:*

**1** **Your** **gender?**

Female

Male

Other. Please clarify:

**2 Your age:**

**3 Your religious belief?**

Catholicism or Christianity

Buddhism or Taoism

Islam

No religion

Other religion. Please clarify:

**4 Your professional specialty?**

Pediatric intensivist

Pediatric hematologist/oncologist

Pediatric neurologist

Pediatric cardiologist

Pediatric anesthetist

General pediatrician

Other. Please clarify:

**5 What is the organizational category of your work setting?**

University hospital

Regional hospital

Community health center

Private clinic/private practice setting

Other. Please clarify:

**6 In what work setting do you work as a pediatrician?**

General pediatric ward

Pediatric intensive care unit

Outpatient setting/private practice space

Other. Please clarify:

**7 How do you spend the majority of your time in your current work setting?**

Direct patient care

Medical management

Medical education

Medical research

**8 What is your current employment status?**

Full-time

Part-time

**9** **How long have you worked as a pediatrician (years)?**

**10** **Have you received formal education in pediatric palliative care?**

Yes

No

Currently undertaking

**11 Do you have experience with caring for dying children?**

Yes

No

**12** **Do you have experience with providing palliative care?**

Yes

No

###### *The Pediatric Palliative Care Attitude Scale*

###### *(PPCAS)*

**Section Two:**

*In the following statements, we will ask you about your perceptions of palliative care practice in pediatrics. To what extent do you disagree or agree with these statements?*

(**Please circle your answer**)

|  | ⯆ | ⯆ | ⯆ | ⯆ | ⯆ |
| --- | --- | --- | --- | --- | --- |
| 1 Palliative care is as important as curative care in the pediatric environment | *Strongly*  *Disagree* | *Somewhat*  *Disagree* | *Somewhat*  *Agree* | *Strongly Agree* | *Unsure* |
| 2 I have had experience of providing palliative care to dying children and their families | *Strongly*  *Disagree* | *Somewhat*  *Disagree* | *Somewhat*  *Agree* | *Strongly Agree* | *Unsure* |
| 3 I feel a sense of personal failure when a child dies | *Strongly*  *Disagree* | *Somewhat*  *Disagree* | *Somewhat*  *Agree* | *Strongly Agree* | *Unsure* |
| 4 There is support for pediatric palliative care in society | *Strongly*  *Disagree* | *Somewhat*  *Disagree* | *Somewhat*  *Agree* | *Strongly Agree* | *Unsure* |
| 5 The medical staff supports palliative care for dying children in my work setting | *Strongly*  *Disagree* | *Somewhat*  *Disagree* | *Somewhat*  *Agree* | *Strongly Agree* | *Unsure* |
| 6 The physical environment of my work setting is ideal for providing palliative care to dying children | *Strongly*  *Disagree* | *Somewhat*  *Disagree* | *Somewhat*  *Agree* | *Strongly Agree* | *Unsure* |
| 7 My work setting is adequately staffed for providing the needs of dying children requiring palliative care and their families | *Strongly*  *Disagree* | *Somewhat*  *Disagree* | *Somewhat*  *Agree* | *Strongly Agree* | *Unsure* |
| 8 In my work setting, parents are involved in decisions about their dying child | *Strongly*  *Disagree* | *Somewhat*  *Disagree* | *Somewhat*  *Agree* | *Strongly Agree* | *Unsure* |
|  | ⯆ | ⯆ | ⯆ | ⯆ | ⯆ |
| 9 My previous experiences of providing palliative care to dying children have been rewarding | *Strongly*  *Disagree* | *Somewhat*  *Disagree* | *Somewhat*  *Agree* | *Strongly Agree* | *Unsure* |
| 10 When children are dying in my work setting, providing pain relief is a priority for me | *Strongly*  *Disagree* | *Somewhat*  *Disagree* | *Somewhat*  *Agree* | *Strongly Agree* | *Unsure* |
| 11 I am often exposed to death in the pediatric environment | *Strongly*  *Disagree* | *Somewhat*  *Disagree* | *Somewhat*  *Agree* | *Strongly Agree* | *Unsure* |
| 12 Palliative care is necessary in pediatric education | *Strongly*  *Disagree* | *Somewhat*  *Disagree* | *Somewhat*  *Agree* | *Strongly Agree* | *Unsure* |
| 13 When a child dies in my work setting, I have sufficient time to spend with the family | *Strongly*  *Disagree* | *Somewhat*  *Disagree* | *Somewhat*  *Agree* | *Strongly Agree* | *Unsure* |
| 14 There are policies/guidelines to assist in the delivery of palliative care in my work setting | *Strongly*  *Disagree* | *Somewhat*  *Disagree* | *Somewhat*  *Agree* | *Strongly Agree* | *Unsure* |
| 15 In my work setting, when a diagnosis with a likely poor outcome is made, parents are informed of palliative care options | *Strongly*  *Disagree* | *Somewhat*  *Disagree* | *Somewhat*  *Agree* | *Strongly Agree* | *Unsure* |
| 16 In my work setting, the team expresses its opinions, values, and beliefs about providing care to dying children | *Strongly*  *Disagree* | *Somewhat*  *Disagree* | *Somewhat*  *Agree* | *Strongly Agree* | *Unsure* |
| 17 Caring for dying children is traumatic for me | *Strongly*  *Disagree* | *Somewhat*  *Disagree* | *Somewhat*  *Agree* | *Strongly Agree* | *Unsure* |
|  | ⯆ | ⯆ | ⯆ | ⯆ | ⯆ |
| 18 I have received in-service education that assists me to support and communicate with parents of dying children | *Strongly*  *Disagree* | *Somewhat*  *Disagree* | *Somewhat*  *Agree* | *Strongly Agree* | *Unsure* |
| 19 All members of the healthcare team in my work setting agree with and support palliative care when it is implemented for a dying child | *Strongly*  *Disagree* | *Somewhat*  *Disagree* | *Somewhat*  *Agree* | *Strongly Agree* | *Unsure* |
| 20 In my work setting, the staff go beyond what they feel comfortable with in using technological life support | *Strongly*  *Disagree* | *Somewhat*  *Disagree* | *Somewhat*  *Agree* | *Strongly Agree* | *Unsure* |
| 21 In my work setting, staff are asked by parents to continue life-extending care beyond what they feel is right | *Strongly*  *Disagree* | *Somewhat*  *Disagree* | *Somewhat*  *Agree* | *Strongly Agree* | *Unsure* |
| 22 My personal attitude about death affects my willingness to deliver palliative care | *Strongly*  *Disagree* | *Somewhat*  *Disagree* | *Somewhat*  *Agree* | *Strongly Agree* | *Unsure* |
| 23 Palliative care is against the values of pediatric medicine | *Strongly*  *Disagree* | *Somewhat*  *Disagree* | *Somewhat*  *Agree* | *Strongly Agree* | *Unsure* |
| 24 When a child dies in my work setting, counselling is available if I need it | *Strongly*  *Disagree* | *Somewhat*  *Disagree* | *Somewhat*  *Agree* | *Strongly Agree* | *Unsure* |
| 25 There is a belief in society that children should not die under any circumstances | *Strongly*  *Disagree* | *Somewhat*  *Disagree* | *Somewhat*  *Agree* | *Strongly Agree* | *Unsure* |
| 26 Curative care is more important than palliative care in the pediatric intensive care environment | *Strongly*  *Disagree* | *Somewhat*  *Disagree* | *Somewhat*  *Agree* | *Strongly Agree* | *Unsure* |

The ‘‘unsure’’ option is positioned in the far right to discourage participants from habitually choosing it.

***Thank you!***

**简体中文版**

**儿童舒缓疗护态度量表**

**第一部分:**

*首先，我们想了解您的以下背景资料：*

**1** **您的性别是？**

女性

男性

其他。请说明:

**2 您的年龄是？**

**3 您的宗教信仰是？**

天主教或基督教

佛教或道教

伊斯兰教

无宗教信仰

其他宗教。请说明:

**4 您的专业方向是?**

儿科重症监护医师

儿科血液学/肿瘤学医师

儿科神经学医师

儿科心脏病学医师

儿科麻醉学医师

全科儿科学医师

其他。请说明:

**5 您的工作单位类别是什么?**

大学附属医院

地区医院

社区保健中心

私人诊所/私人执业机构

其他（如私立医院）。请说明:

**6 您在什么科室担任儿科医生?**

普通儿科

儿科重症监护室

门诊/私人执业点

其他。请说明:

**7 您目前的工作状态是?**

全职

兼职

**8 您目前的主要工作内容是?**

临床实践

医疗管理

医学教育

医学研究

**9** **您担任儿科医生的年限是?**

**10** **您是否接受过儿童舒缓疗护的专业课程/培训？**

是

否

正在接受

**11 您是否有治疗临终患儿的经验?**

是

否

**12** **您是否有提供舒缓疗护的经验？**

是

否

###### 儿童舒缓疗护态度量表

**第二部分:**

*在以下条目中，我们将询问您对儿童舒缓疗护的看法。请回答您在多大程度上同意或不同意这些条目？*

(**请圈出您的回答**)

|  | ⯆ | ⯆ | ⯆ | ⯆ | ⯆ |
| --- | --- | --- | --- | --- | --- |
| 1 在儿科，舒缓疗护跟医学治疗一样重要 | *非常不同意* | *不太同意* | *比较同意* | *非常同意* | *不确定* |
| 2 我有为临终患儿及他们家人提供舒缓疗护的经验 | *非常不同意* | *不太同意* | *比较同意* | *非常同意* | *不确定* |
| 3 当有患儿死亡时，我会有挫败感 | *非常不同意* | *不太同意* | *比较同意* | *非常同意* | *不确定* |
| 4 我相信社会上的大多数人都同意儿童舒缓疗护的观点 | *非常不同意* | *不太同意* | *比较同意* | *非常同意* | *不确定* |
| 5 在我的工作单位，医护人员支持为临终患儿提供舒缓疗护 | *非常不同意* | *不太同意* | *比较同意* | *非常同意* | *不确定* |
| 6 我工作单位的设备可为临终患儿提供理想的舒缓疗护环境 | *非常不同意* | *不太同意* | *比较同意* | *非常同意* | *不确定* |
| 7 我的工作单位有充足的医护人员，能为临终患儿及其家属提供所需的舒缓疗护 | *非常不同意* | *不太同意* | *比较同意* | *非常同意* | *不确定* |
| 8 在我的工作单位，父母能参与与临终患儿相关的决定 | *非常不同意* | *不太同意* | *比较同意* | *非常同意* | *不确定* |
|  | ⯆ | ⯆ | ⯆ | ⯆ | ⯆ |
| 9 我对过去提供舒缓疗护给临终患儿的经验感到满意 | *非常不同意* | *不太同意* | *比较同意* | *非常同意* | *不确定* |
| 10 我认为面对临终患儿时，缓解疼痛是我的首要任务 | *非常不同意* | *不太同意* | *比较同意* | *非常同意* | *不确定* |
| 11 在我的工作单位，我经常面对死亡 | *非常不同意* | *不太同意* | *比较同意* | *非常同意* | *不确定* |
| 12 儿科医护教育有必要包括舒缓疗护 | *非常不同意* | *不太同意* | *比较同意* | *非常同意* | *不确定* |
| 13 在我的工作单位里若患儿死亡，我有充足的时间陪伴家属 | *非常不同意* | *不太同意* | *比较同意* | *非常同意* | *不确定* |
| 14 我的工作单位有指导实施舒缓疗护的政策或指南 | *非常不同意* | *不太同意* | *比较同意* | *非常同意* | *不确定* |
| 15 在我的工作单位，若患儿被诊断预后较差，医护人员会告知父母舒缓疗护的概念 | *非常不同意* | *不太同意* | *比较同意* | *非常同意* | *不确定* |
| 16 在我的工作单位，舒缓疗护团队可以为向临终患儿提供舒缓疗护表达各自的意见、价值观或想法 | *非常不同意* | *不太同意* | *比较同意* | *非常同意* | *不确定* |
| 17 照护临终患儿给我带来痛苦的感受 | *非常不同意* | *不太同意* | *比较同意* | *非常同意* | *不确定* |
| 18 我接受过相关的教育和培训，足以让我为临终患儿的父母提供支持，也能与他们无障碍地沟通 | *非常不同意* | *不太同意* | *比较同意* | *非常同意* | *不确定* |
|  | ⯆ | ⯆ | ⯆ | ⯆ | ⯆ |
| 19 向临终患儿提舒缓疗护时，我所在工作单位的医护人员均同意且支持这个做法 | *非常不同意* | *不太同意* | *比较同意* | *非常同意* | *不确定* |
| 20 在我的工作单位，医护人员即使觉得不妥当，仍会使用仪器维持患儿的生命 | *非常不同意* | *不太同意* | *比较同意* | *非常同意* | *不确定* |
| 21 在我的工作单位，即使医护人员不认同维持生命治疗，但在父母要求下，仍会配合进行 | *非常不同意* | *不太同意* | *比较同意* | *非常同意* | *不确定* |
| 22 我对死亡的态度常影响我提供舒缓疗护的意愿 | *非常不同意* | *不太同意* | *比较同意* | *非常同意* | *不确定* |
| 23 舒缓疗护违反了儿科治疗的价值观 | *非常不同意* | *不太同意* | *比较同意* | *非常同意* | *不确定* |
| 24 在我的工作单位如有患儿死亡，若我有需要，可咨询其他同事 | *非常不同意* | *不太同意* | *比较同意* | *非常同意* | *不确定* |
| 25 社会上有一种信念，认为无论发生什么状况，患儿都不该死亡 | *非常不同意* | *不太同意* | *比较同意* | *非常同意* | *不确定* |
| 26 在儿科重症监护室，医学治疗比舒缓疗护重要 | *非常不同意* | *不太同意* | *比较同意* | *非常同意* | *不确定* |

***感谢您的参与!***
