# Supplementary material for: Chinese and Belgian pediatricians’ perspectives toward pediatric palliative care: an online survey
Source: BMC Palliat Care. 2024 Apr 23;23:106. doi: 10.1186/s12904-024-01436-0 (PMC11036583; doi:10.1186/s12904-024-01436-0)
Supplement: Supplementary file 3 — Supplementary Material 3 [file 12904_2024_1436_MOESM3_ESM.docx]

**Supplementary Material 3. Rotated Factors Matrix for Final EFA^a^ of the PPCAS (n=440)**

| **22 Items^b^** | **Factor loadings^c^** | | | | **Communities** |
| --- | --- | --- | --- | --- | --- |
|  | **F1** | **F2** | **F3** | **F4** |  |
| 6. The physical environment of my work setting is ideal for providing palliative care to dying children | **.781** | -.200 | -.046 | .020 | .653 |
| 7. My work setting is adequately staffed for providing the needs of dying children requiring palliative care and their families | **.780** | -.167 | -.054 | .068 | .644 |
| 14. There are policies/guidelines to assist in the delivery of palliative care in my work setting | **.761** | .024 | -.016 | .240 | .637 |
| 16. In my work setting, the team expresses its opinions, values, and beliefs about providing care to dying children | **.712** | .240 | .166 | .266 | .663 |
| 5. The medical staff supports palliative care for dying children in my work setting | **.672** | -.052 | .360 | .012 | .583 |
| 15. In my work setting, when a diagnosis with a likely poor outcome is made, parents are informed of palliative care options | **.638** | .281 | .157 | .283 | .591 |
| 13. When a child dies in my work setting, I have sufficient time to spend with the family | **.529** | .276 | .041 | **.443** | .553 |
| 9. My previous experiences of providing palliative care to dying children have been rewarding | **.529** | .228 | .127 | **.496** | .594 |
| 19. All members of the healthcare team in my work setting agree with and support palliative care when it is implemented for a dying child | **.497** | .062 | .391 | .184 | .438 |
| 21. In my work setting, staff are asked by parents to continue life-extending care beyond what they feel is right | .060 | **.737** | -.104 | -.058 | .561 |
| 3. I feel a sense of personal failure when a child dies | .039 | **.696** | .197 | .268 | .597 |
| 20. In my work setting, the staff go beyond what they feel comfortable with in using technological life support | .102 | **.689** | -.052 | -.156 | .513 |
| 17. Caring for dying children is traumatic for me | -.063 | **.581** | .046 | .096 | .353 |
| 22. My personal attitude about death affects my willingness to deliver palliative care | -.016 | **.555** | .082 | -.083 | .322 |
| 26. Curative care is more important than palliative care in the pediatric intensive care environment | .014 | **.457** | .253 | -.037 | .274 |
| 12. Palliative care is necessary in pediatric education | -.011 | -.029 | **.761** | .080 | .587 |
| 10. When children are dying in my work setting, providing pain relief is a priority for me | .079 | .102 | **.616** | .213 | .441 |
| 23. Palliative care is against the values of pediatric medicine | -.090 | .391 | **.583** | -.278 | .578 |
| 1. Palliative care is as important as curative care in the pediatric environment | .244 | .033 | **.526** | .008 | .337 |
| 2. I have had experience of providing palliative care to dying children and their families | .274 | .122 | .173 | **.683** | .587 |
| 11. I am often exposed to death in the pediatric environment | .086 | -.307 | .076 | **.568** | .430 |
| 18. I have received in-service education that assists me to support and communicate with parents of dying children | .396 | -.216 | -.113 | **.555** | .524 |
| Eigenvalue | 25.261 | 14.289 | 7.461 | 5.085 | NA |
| % of variance explained | 19.557 | 13.722 | 9.573 | 9.244 | NA |

Extraction: Principal Component Analysis; Rotation: Varimax with Kaiser Normalization.

Abbreviations: EFA=exploratory factor analysis; PPCAS=Pediatric Palliative Care Attitude Scale; NA=not applicable.

^a^ The Kaiser-Meyer-Oklin value was 0.86 (over recommended value of 0.60); the Bartlett’s Test of Sphericity was 0.000, indicating that the data were suitable for conducting meaningful EFA

^b^The EFA produced a five-factor model, but we removed one factor (comprising items 4, 8, 24, and 25), because its Cronbach’s α was 0.36, indicating that the factor was unreliable (i.e., was not a theoretically meaningful factor). Therefore, only 22 of the 26 PPCAS items are shown above.

^c^Factor loading values ≥0.4 are in bold.
