# Supplementary material for: Chinese and Belgian pediatricians’ perspectives toward pediatric palliative care: an online survey
Source: BMC Palliat Care. 2024 Apr 23;23:106. doi: 10.1186/s12904-024-01436-0 (PMC11036583; doi:10.1186/s12904-024-01436-0)
Supplement: Supplementary file 4 — Supplementary Material 4 [file 12904_2024_1436_MOESM4_ESM.docx]

**Supplementary Material 4. Comparison of Mainland Chinese Pediatricians’ Characteristics and Mean Subscale Scores (n=325)**

| **Variable** |  | **Unit Support**  **Mean±SD** | **Test Statistics** | ***p*-value^a^** | **Personal Obstacles**  **Mean±SD** | **Test Statistics** | ***p*-value^a^** | **PPC Importance**  **Mean±SD** | **Test Statistics** | ***p*-value^a^** | **Work Experience**  **Mean±SD** | **Test Statistics** | ***p*-value^a^** |
| --- | --- | --- | --- | --- | --- | --- | --- | --- | --- | --- | --- | --- | --- |
| **Gender** | Female | 2.86±0.84 | t=-0.484 | 0.629 | 2.27±0.59 | t=0.947 | 0.344 | 3.91±0.49 | t=1.906 | 0.057 | 2.70±0.94 | t=-1.751 | 0.081 |
|  | Male | 2.91±0.86 |  |  | 2.20±0.55 |  |  | 3.79±0.56 |  |  | 2.88±0.88 |  |  |
| **Age** |  | 2.88±0.85 | ρ=-0.048 | 0.390 | 2.25±0.58 | ρ=-0.098 | 0.077 | 3.87±0.52 | ρ=0.170 | **0.002** | 2.76±0.92 | ρ=0.023 | 0.686 |
| **Religious beliefs** | No religion | 2.89±0.86 | t=0.501 | 0.617 | 2.24±0.57 | t=-0.673 | 0.501 | 3.88±0.51 | t=1.156 | 0.248 | 2.75±0.93 | t=-0.494 | 0.622 |
|  | Have religious beliefs | 2.81±0.80 |  |  | 2.32±0.65 |  |  | 3.77±0.57 |  |  | 2.84±0.82 |  |  |
| **Professional specialty** | General pediatrician | 2.84±0.87 | t=-0.862 | 0.389 | 2.27±0.59 | t=0.531 | 0.596 | 3.82±0.52 | t=-1.880 | 0.061 | 2.64±0.92 | t=-2.469 | **0.014** |
|  | Pediatric specialist | 2.92±0.82 |  |  | 2.23±0.57 |  |  | 3.93±0.51 |  |  | 2.89±0.91 |  |  |
| **Institutional setting** | University hospital | 2.92±0.81 | t=0.968 | 0.334 | 2.24±0.56 | t=-0.223 | 0.824 | 3.87±0.50 | t=-0.091 | 0.928 | 2.76±0.91 | t=-0.040 | 0.968 |
|  | Regional hospital and other setting | 2.83±0.89 |  |  | 2.26±0.60 |  |  | 3.87±0.53 |  |  | 2.76±0.94 |  |  |
| **Work department** | General pediatric ward and private practice space | 2.86±0.88 | t=-0.687 | 0.493 | 2.24±0.57 | t=-0.268 | 0.789 | 3.84±0.53 | t=-1.821 | 0.070 | 2.67±0.94 | t=-3,715 | **<0.001** |
|  | Pediatric subspecialty ward | 2.94±0.72 |  |  | 2.27±0.63 |  |  | 3.97±0.45 |  |  | 3.08±0.78 |  |  |
| **Main work** | Direct patient care | 2.87±0.85 | t=-0.733 | 0.464 | 2.26±0.58 | t=0.965 | 0.335 | 3.87±0.51 | t=0.354 | 0.362 | 2.75±0.92 | t=-0.716 | 0.475 |
|  | Medical management, research, or education | 3.03±0.90 |  |  | 2.11±0.58 |  |  | 3.83±0.57 |  |  | 2.92±1.04 |  |  |
| **Employment status** | Full-time | 2.88±0.85 | t=-0.169 | 0.866 | 2.25±0.58 | t=0.351 | 0.726 | 3.87±0.51 | t=0.973 | 0.331 | 2.76±0.92 | t=-0.529 | 0.597 |
|  | Part-time | 2.94±0.81 |  |  | 2.17±0.64 |  |  | 3.67±0.56 |  |  | 2.96±0.91 |  |  |
| **Years of being a pediatrician** |  | 2.88±0.85 | ρ=-0.072 | 0.195 | 2.25±0.58 | ρ=-0.043 | 0.437 | 3.87±0.52 | ρ=0.133 | **0.016** | 2.76±0.92 | ρ=-0.005 | 0.925 |
| **Received PPC education** | Yes | 3.76±0.77 | t=-7.297 | **<0.001** | 2.26±0.66 | t=0.157 | 0.875 | 3.84±0.57 | t=-0.415 | 0.678 | 3.82±0.86 | t=8.241 | **<0.001** |
|  | No | 2.76±0.79 |  |  | 2.25±0.57 |  |  | 3.87±0.51 |  |  | 2.62±0.84 |  |  |
| **Experience caring for dying children** | Yes | 3.02±0.86 | t=2.254 | **0.025** | 2.23±0.61 | t=-0.478 | 0.633 | 3.96±0.52 | t=2.341 | **0.020** | 3.16±0.91 | t=6.205 | **<0.001** |
|  | No | 2.80±0.84 |  |  | 2.26±0.57 |  |  | 3.82±0.51 |  |  | 2.53±0.85 |  |  |
| **Experience providing PPC** | Yes | 3.49±0.77 | t=4.930 | **<0.001** | 2.26±0.56 | t=0.082 | 0.935 | 4.00±0.55 | t=1.689 | 0.092 | 3.58±0.91 | t=6.223 | **<0.001** |
|  | No | 2.80±0.83 |  |  | 2.25±0.58 |  |  | 3.85±0.51 |  |  | 2.65±0.88 |  |  |

Abbreviations: SD=standard deviation; t=independent t-test; ρ=Spearman correlation coefficient.

^a^*p*-value: Comparison between pediatricians’ characteristics and mean subscale scores; <0.05 was considered statistically significant (in bold).
