# Supplementary material for: Chinese and Belgian pediatricians’ perspectives toward pediatric palliative care: an online survey
Source: BMC Palliat Care. 2024 Apr 23;23:106. doi: 10.1186/s12904-024-01436-0 (PMC11036583; doi:10.1186/s12904-024-01436-0)
Supplement: Supplementary file 5 — Supplementary Material 5 [file 12904_2024_1436_MOESM5_ESM.docx]

**Supplementary Material 5. Comparison of Flemish Pediatricians’ Characteristics and Mean^a^ or Median^b^ Subscale Scores (n=115)**

| **Variable** |  | **Unit Support**  **Mean±SD** | **Test statistics** | ***p*-value^c^** | **Personal Obstacles**  **Mean±SD** | **Test statistics** | ***p*-value^c^** | **PPC Importance**  **Mean±SD/**  **Median (Q1-Q3)** | **Test statistics** | ***p*-value^c^** | **Work Experience**  **Mean±SD** | **Test statistics** | ***p*-value^c^** |
| --- | --- | --- | --- | --- | --- | --- | --- | --- | --- | --- | --- | --- | --- |
| **Gender** | Female | 3.43±0.86 | t=0.134 | 0.894 | 3.51±0.75 | t=0.078 | 0.938 | 4.60  (4.20-4.80) | U=  1123.500^g^ | 0.820 | 2.90±0.94 | t=-0.401 | 0.689 |
|  | Male | 3.40±0.88 |  |  | 3.49±0.81 |  |  | 4.80  (4.00-5.00) |  |  | 2.98±0.97 |  |  |
| **Age** |  | 3.42±0.86 | ρ=0.209 | **0.025** | 3.50±0.76 | ρ=-0.012 | 0.895 | 4.51±0.46 | ρ=0.057 | 0.547 | 2.92±0.94 | ρ=0.226 | **0.015** |
| **Religious beliefs** | No religion | 3.40±0.83 | t=-0.215 | 0.830 | 3.56±0.79 | t=0.732 | 0.466 | 4.80  (4.20-5.00) | U=  1548.500 | 0.632 | 3.00±0.96 | t=0.810 | 0.420 |
|  | Have religious beliefs | 3.44±0.89 |  |  | 3.46±0.74 |  |  | 4.60  (4.20-4.80) |  |  | 2.85±0.93 |  |  |
| **Professional specialty** | General pediatrician | 3.10±0.84 | t=-5.132 | **<0.001** | 3.47±0.76 | t=-0.511 | 0.610 | 4.60  (4.20-4.80) | U=  1378.000 | 0.156 | 2.50±0.78 | t=-6.207 | **<0.001** |
|  | Pediatric specialist | 3.85±0.70 |  |  | 3.54±0.76 |  |  | 4.80  (4.20-5.00) |  |  | 3.46±0.87 |  |  |
| **Institutional setting** | University hospital | 4.01±0.72 | t=6.785 | **<0.001** | 3.42±0.76 | t=-0.887 | 0.377 | 4.80  (4.20-4.95) | U=  1355.500 | 0.227 | 3.51±0.89 | t=6.058 | **<0.001** |
|  | Regional hospital and other setting | 3.06±0.74 |  |  | 3.55±0.76 |  |  | 4.60  (4.20-4.80) |  |  | 2.55±0.78 |  |  |
| **Work department** | General pediatric ward, and private practice space | 3.39±0.88 | t=-1.202 | 0.232 | 3.48±0.76 | t=-0.693 | 0.489 | 4.80  (4.20-4.80) | U=  769.000 | 0.850 | 2.79±0.91 | t=-3.895 | **<0.001** |
|  | Pediatric subspecialty ward | 3.66±0.75 |  |  | 3.63±0.76 |  |  | 4.50  (4.20-5.00) |  |  | 3.72±0.73 |  |  |
| **Employment status** | Full-time | 3.39±0.86 | t=-1.076 | 0.284 | 3.49±0.78 | t=-0.412 | 0.681 | 4.60  (4.20-4.95) | U=  895.500 | 0.899 | 2.87±0.92 | t=-1.096 | 0.276 |
|  | Part-time | 3.62±0.87 |  |  | 3.57±0.68 |  |  | 4.80  (4.20-4.80) |  |  | 3.13±1.03 |  |  |
| **Years of being pediatrician** |  | 3.42±0.86 | ρ=0.234 | **0.012** | 3.50±0.76 | ρ=-0.12 | 0.898 | 4.51±0.46 | ρ=0.055 | 0.886 | 2.92±0.94 | ρ=0.197 | **0.035** |
| **Received PPC Education** | Yes | 4.00±0.71 | t=2.739 | **0.007** | 3.79±0.73 | t=1.497 | 0.137 | 4.80  (4.55-5.00) | U=  481.500 | 0.050 | 3.91±0.68 | t=4.568 | **<0.001** |
|  | No | 3.34±0.85 |  |  | 3.46±0.76 |  |  | 4.60  (4.20-4.80) |  |  | 2.78±0.89 |  |  |
| **Experience caring for dying children** | Yes | 3.65±0.78 | t=4.715 | **<0.001** | 3.58±0.76 | t=1.763 | 0.081 | 4.80  (4.20-5.00) | U=  1059.500 | **0.048** | 3.28±0.84 | t=8.103 | **<0.001** |
|  | No | 2.89±0.83 |  |  | 3.31±0.73 |  |  | 4.40  (4.00-4.80) |  |  | 2.04±0.47 |  |  |
| **Experience providing PPC** | Yes | 3.71±0.72 | t=4.356 | **<0.001** | 3.57±0.79 | t=0.997 | =0.160 | 4.80  (4.20-5.00) | U=  1325.500 | =0.079 | 3.53±0.70 | t=11.525 | **<0.001** |
|  | No | 3.06±0.89 |  |  | 3.42±0.71 |  |  | 4.40  (4.00-4.80) |  |  | 2.14±0.56 |  |  |
|  |  |  |  |  |  |  |  |  |  |  |  |  |  |

Abbreviations: SD=standard deviation; Q1=first quantile; Q3=third quantile; t=independent t-test; ρ=Spearman correlation coefficient; U=Mann-Whitney U test.

^a^Mean: For normally distributed data.

^b^Median: For non-normally distributed data.

^c^*p*-value: Comparison between pediatricians’ characteristics and mean subscale scores; <0.05 was considered statistically significant (in bold).
